# Topographic Evaluation of Inflammatory Periapical Lesions in the First Molar's Region Using CBCT

**DOI:** 10.1155/ijod/8992304

**Published:** 2025-01-21

**Authors:** Maryam Kazemipoor, Fatemeh Foroughipour, Yaser Safi

**Affiliations:** ^1^Department of Endodontics, School of Dentistry, Shahid Sadoughi University of Medical Sciences, Yazd, Iran; ^2^Department of Oral and Maxillofacial Radiology, School of Dentistry, Shahid Beheshti University of Medical Sciences, Tehran, Iran

**Keywords:** cone-beam computed tomography, diagnosis, endodontics, molars, periapical disease, periapical periodontitis

## Abstract

**Background:** Investigating the pattern of extension in the periapical (PA) inflammatory lesions is important in the treatment plan and prognosis of treatment.

**Introduction:** This study evaluated the topography of PA inflammatory lesions in the first molars using cone-beam computed tomography (CBCT).

**Methods:** In this descriptive study, 197 CBCT images about patients in the age group of 14–77 years were analyzed. The maximum extension of the PA lesion in the three orthogonal planes related to the regions of maxillary and mandibular first molars was measured and reported in millimeters. Measurements were compared based on age, gender, dental arch, and root type. Statistical analysis was performed using percentages, repeated measure ANOVA, paired *t*-tests, and Pearson correlation coefficient. The significant level was set at 0.05.

**Results:** The highest total mean lesion extensions were in the vertical plane followed by the buccolingual and mesiodistal plane. There was a statistically significant difference between the extension of the PA lesion in the vertical and mesiodistal (*p* < 0.001), vertical and buccolingual (*p* =0.001), as well as the mesiodistal and buccolingual planes (*p* =0.027). In the maxilla and mandible, the highest mean lesion extension was in the vertical, buccolingual, and mesiodistal plane, respectively. According to the root type, there was only a statistically significant difference in lesion extension in the buccolingual plane and between the mesial and distobuccal roots (*p* =0.030).

**Conclusion:** Given the limitations of the present study, regarding the extension of the PA lesion in the first molar region, the bone structure of the maxilla and mandible follows a precise and delicate pattern. In this regard, future studies in different communities and races should be designed to address this issue in different communities. In addition, CBCT is a reliable imaging method to evaluate the extension of the PA lesion both morphologically and morphometrically.

## 1. Introduction

Dental pulp is a sterile environment, shielded by enamel, cementum, and most importantly, protected by dentin which constructs a dynamic dentin-pulp complex. Serious damage to these structures leads to pulpitis, and if tooth inflammation continues, it causes necrosis [1]. The effect of these inflammatory processes on the root canal system and periapical (PA) tissue leads to PA bone lesions, which constitute 75% of primary endodontic lesions [2–4]. Although most of these lesions are without clinical symptoms [5], they can affect the treatment and prognosis of root canal therapy (RCT) and future tooth replacement approaches [1, 2, 5, 6]. Histological evaluation, though considered the gold standard for diagnosing PA lesions, is invasive. Consequently, radiography and clinical examinations are the primary methods for diagnosing and evaluating these lesions [2].

Intraoral PA radiographs have long been used for this purpose. However, these lesions only become visible on PA radiographs after 30% to 50% of the bone mineral content has been lost during disease progression [5, 6]. Several factors affect the detection of these lesions in radiographic images, including the density of the surrounding bone, the tooth's location, the lesion's three-dimensional shape, the X-ray projection angle, and image contrast [6]. PA images are two-dimensional, which somewhat limits the information about the lesion's size, extension, and location. Additionally, important structures in RCT may be obscured by anatomical features in these radiographs [5–7]. Today, cone-beam computed tomography (CBCT) is a relatively new method in the diagnosis of PA lesions which could drastically guide an endodontic treatment plan, a retreatment, a retrograde surgery, and finally improving the prognosis greatly [2, 6]. In this type of imaging, the superimposition seen in regular radiographs does not occur and the anatomical structures are seen more clearly. CBCT images with their 3D view can show us the relationship of the lesion with important anatomical structures such as the maxillary sinus and the mandibular canal more precisely [7]. In a comparison between PA radiographs, CBCT images, and histological evaluation, it has been shown that bone lesions in PA radiographs were not detectable in 22% of cases, while this value was 9% in CBCT [2]. Molars are the most difficult teeth for interpreting PA lesions in PA graphs; but when CBCT images were used to diagnose these lesions, the number of detected PA lesions increased by 63% [6]. In the posterior region of the mandible, the molars tend to be lingually inclined, and this causes the roots of these teeth to be in the vicinity of the lingual nerve. Besides, the extension of PA inflammatory lesions in this region has a greater tendency to the lingual area [8]. The proximity of the root tip of the mandibular molar teeth to the inferior alveolar nerve (IAN) canal causes the nerve to be damaged during nonsurgical RCT due to filling behind the canal or during endodontic surgery. In addition, the extension of local infections such as PA inflammatory lesions in this region can cause nerve paresthesia [9, 10]. In maxillary molars, the maxillary sinus is in the vicinity of the molar roots and this proximity can cause the extension of PA inflammatory lesions to the sinus space [11]. Considering the proximity of molars to important anatomical structures and the inefficacy of two-dimensional PA radiographs in determining the pattern of PA lesion extension in this region, the investigation of the PA lesion and the pattern of bone destruction with three-dimensional imaging methods is of utmost importance. Hence, the present study aimed to investigate the pattern of extension in PA inflammatory lesions attributed to maxillary and mandibular first molars in the three spatial planes of axial, coronal, and sagittal using CBCT.

## 2. Material and Methods

The protocol of this study was approved by the Shahid Sadoughi University of Medical Sciences Ethics Committee Yazd, Iran (IR.SSU.REC.1400.004) before data analysis. The inclusion criteria for the study were specified as maxillary and mandibular molar teeth with PA lesions, the age of the patients in the range of 14–77 years, and teeth with fully formed apices. Regarding the most prevalent root configurations for maxillary and mandibular molars, which typically consist of three roots (one palatal and two buccal roots) or two roots (mesial and distal), respectively, and to maintain consistency, all variations outside these common anatomical norms excluded from the study sample. Radiologically, PA lesions were characterized as radiolucent areas or dark regions observed in proximity to the root apex when examining CBCT scans. The primary objective of the present study was to evaluate the extension of inflammatory PA lesions. To achieve accurate diagnoses, a multifaceted diagnostic approach was applied which aimed to distinguish between anatomical, developmental, and inflammatory radiolucency. Given the higher prevalence of inflammatory PA lesions, the specific criteria to differentiate between inflammatory and noninflammatory PA lesions were as follows:

1. Proximity and merging view: Considering the location of bone loss in the PA region, particularly the proximity of the root apex within the lesion.
2. Lamina dura disruption: Considering the disruption of the lamina dura, a characteristic more frequently associated with inflammatory lesions.
3. Size and shape: The dimensions and shape of the lesion were carefully assessed to aid in the differentiation process.
4. Tooth evaluation: Thoroughly evaluated the affected teeth and investigated potential factors contributing to the PA lesion.

Each subject had buccal or lingual–palatal plates perforation and open apices were excluded from the analysis.

In this descriptive-correlational study, the research samples were 197 CBCT images stored in the archives of a private radiology center in Tehran. CBCT scans were captured by Scanora 3D (Soredex, Tuusula, Finland) with exposure settings of 13 mA, 90 kVp, scan/exposure time of 16/3.75 s, a voxel size of 0.20, and 0.5 mm slice thickness. Information about each patient including sex, age, dental arch, and root type recorded in special tables designed for the present study.

The samples included 84 (43.2%) men and 113 (56.8%) women aged 14–77 years who were randomly selected from the available data set. The 197 images included maxillary and mandibular first molars with PA lesions selected from 812 CBCTs. Samples were categorized into three age groups: A (14–44 years), B (45–54 years), and C (55–77 years).

CBCT images were evaluated using a computer (Lenovo IdeaPad 310-15IKB, IBM Co., USA) with a screen size of 15.40 and a resolution of 1280 × 800 and RadiAnt software (RadiAnt DICOM Viewer, Radiant Software Solution Inc., Maharashtra, India).

**Figure 1:**
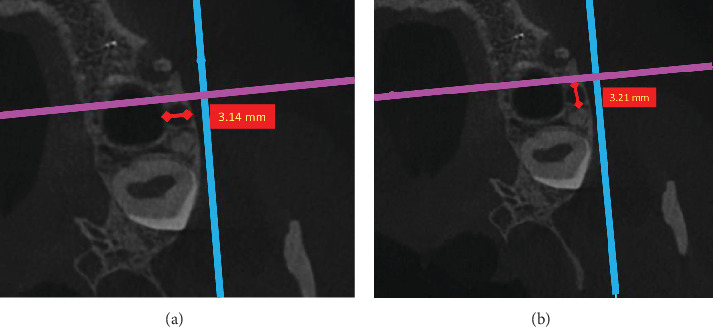
Periapical lesion extension in axial view: (a) buccolingual dimension and (b) mesiodistal dimension.

A manual magnifier with a magnification of 2.5X and the internal zoom of the software was applied for magnifying, increasing the accuracy, and visibility of the lesions' border. The lesions' border was determined by the naked eye. Both an endodontist and an oral radiologist conducted all measurements simultaneously. In the axial plane, maximum lesion extensions in the horizontal buccolingual (buccopalatal) and horizontal mesiodistal dimensions were measured in millimeters using a software ruler (Figure 1a,b).

**Figure 2:**
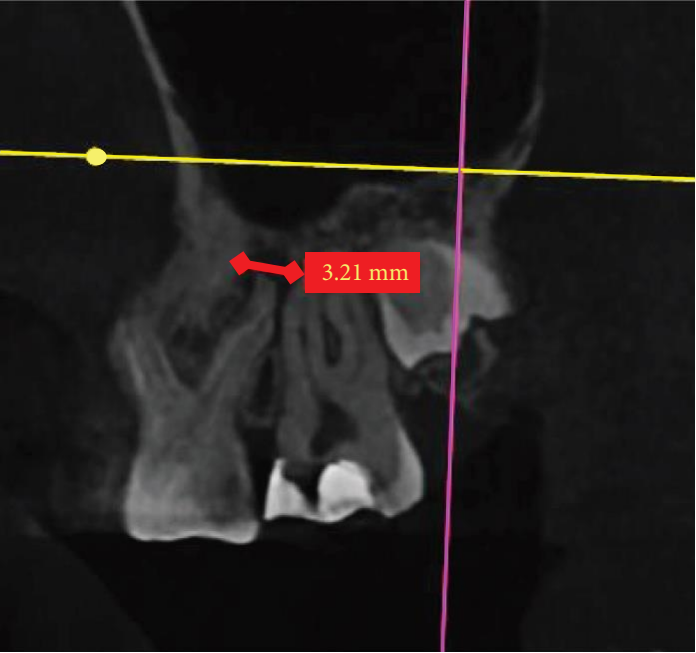
Periapical lesion extension in the sagittal view.

**Figure 3:**
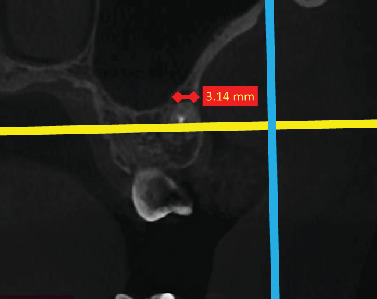
Periapical lesion extension in the coronal view.

In the sagittal plane, the lesion's maximum vertical (occluso-apical) and horizontal mesiodistal extensions were measured (Figure 2). In the same manner, in the coronal plane, the maximum vertical (occluso-apical) and horizontal buccolingual (buccopalatal) extensions of the lesion were recorded (Figure 3). The highest rate of lesion extension was reported for each root of the first molars. To ensure consistent and homogeneous evaluation of inflammatory PA lesions, we adopted a uniform approach. Our strategy involved tracing the axial view to identify the maximum extensions in both horizontal planes. Subsequently, we conducted measurements on this plane. To validate our measurements, we cross-confirmed them in the coronal and sagittal planes.

When it came to vertical measurements, we utilized the coronal and sagittal planes for confirmation. This choice was made because the long axis of the tooth may change concerning the planes, but the maximum border of the lesion remained consistent and unchanged.

Data were recorded in SPSS software (SPSS version 24, Chicago, IL, USA). Statistical analysis was performed using percentages, repeated measures ANOVA, paired *t*-test, and Pearson's correlation coefficient. The significant level was set at 0.05.

## 3. Results

Repeated measures ANOVA showed that the extensions of the lesion were not similar in all directions and there were significant differences between the lesion expansions in the three-dimensional planes (*p* < 0.001). Generally, in the examined population, the highest average of lesion extension was reported in the vertical dimension (2.04 ± 1.61), followed by horizontal buccolingual (1.84 ± 1.28) and horizontal mesiodistal dimension (1.74 ± 1.19), respectively (Table 1).

A statistically significant difference was reported between the lesion extension in vertical and buccolingual (*p*=0.001), vertical and mesiodistal (*p* < 0.001), and between two horizontal planes (*p*=0.027). In the age group of 14–44 years, the highest mean lesion extension was observed in the vertical, buccolingual, and mesiodistal planes, respectively. In the age group of 45–54 years, the highest mean lesion extension was observed in the vertical, mesiodistal, and buccolingual planes, respectively. Also, in the age group of 55–77 years, the highest mean lesion extension was observed in the buccolingual aspect, vertical aspect, and mesiodistal planes, respectively. Regarding the effect of age, a statistically significant difference was reported in lesion extension in the vertical (*p*=0.015) and mesiodistal (*p*=0.034) planes between the age groups of 14–44 and 55–77 years (Table 1). A different pattern of lesion extension was reported between men (*n* = 84) and women (*n* = 113). In the male patients, the highest mean lesion extension was observed in the vertical, buccolingual, and mesiodistal planes, respectively. In contrast in the female patients, the highest mean lesion extension was observed in the vertical, mesiodistal, and buccolingual aspects, respectively. No statistically significant difference was reported between men and women with regard to lesion extension (Table 2). According to the dental arch, 61.42% of the samples were related to the maxilla and 38.57% were related to the mandible. A similar extension pattern was reported for both the maxilla and mandible. The highest mean lesion extension in either maxilla or mandible was reported in the vertical, buccolingual, and mesiodistal, respectively. No statistically significant difference was reported between the two jaws regarding lesion extension (Table 3). Two hundred eighty-nine lesions were reported in the present study, which included 13.84% distal roots, 22.14% mesial roots, 13.84% distobuccal roots, 32.87% mesiobuccal roots, and 17.3% palatal roots. The highest mean lesion extension in all the molars' roots was reported in the vertical plane. In the distal, distobuccal, and mesiobuccal roots, the highest mean lesion extension was related to the vertical, buccolingual, and mesiodistal, respectively. In the mesial and palatal roots, the highest mean lesion extension was related to the vertical, mesiodistal, and buccolingual planes. Regarding the effect of root type, there was only a statistically significant difference in the buccolingual plane (*p*=0.024). A pairwise comparison of the two groups revealed that there was only a statistically significant difference between the buccolingual extension of the mesial root of mandibular molars and the distobuccal root of the maxillary molars (*p*=0.030) (Table 4).

## 4. Discussion

Microorganisms and their secondary byproducts are central to the initiation and progression of pulpal diseases, which extend into the PA tissue [12]. The success of RCT depends on thoroughly eliminating these microorganisms from the root canal system [12]. Traditionally, intraoral PA radiographs are used to assess RCT's success [13]. During long-term follow-up, the healing or progression of PA lesions linked to root canal treatment is a key factor in determining success [1]. Given that intraoral PA radiographs provide a two-dimensional view, it is recommended to take at least two radiographs with different projection angles to better assess the lesion's third dimension [14]. CBCT imaging, highly accurate for early diagnosis of PA diseases, can reveal the lesion's actual location [15]. The base of the maxillary sinus extends from the first premolar region to the maxillary tuberosity. In some cases, the roots of the posterior maxillary teeth are close to the sinus floor, allowing odontogenic infections in the PA area to spread to the maxillary sinus and cause maxillary sinusitis [16].

Previous studies have concluded that CBCT images detect PA lesions in the upper jaw 34% more than PA images. Besides, CBCT images can show the extension of the lesion to the sinus [16]. In a study aimed at the anatomical analysis of the PA bone of the maxillary posterior teeth with CBCT, Hu et al. [16] concluded that in the upper posterior teeth, the mesiobuccal root of the upper second molar is located at the closest distance to the sinus floor and the greatest vertical distance can be seen in the buccal roots of the first maxillary premolar. They also demonstrated that this distance is also related to age and sex, and this gap was reported more in people over 40 years old and women [16]. During research conducted in 2020, Sakir and Ercalik Yalcinkaya [17] concluded that the thickness of the sinus mucosa can be evaluated in three-dimensional images such as CBCT compared to two-dimensional images. The thickness of the sinus mucosa is greater when the roots of the molars are closer to the sinus floor and have a PA lesion [17]. Since CBCT images can determine the thickness and density of bone, the inclination of roots, and their relationship with anatomical structures, thus, it is necessary to measure the real topography of the PA lesion with CBCT images before surgical procedures [18]. Meirinhos et al. [19] investigated the relationship between the prevalence of PA lesions with previous RCT and the type of crown restoration with the help of CBCT during a cross-sectional study. They concluded that maxillary first molars have a higher prevalence of PA lesions. In terms of roots, the mesiobuccal roots of maxillary first molars had the highest prevalence of lesions [19]. In a study aimed at determining the prevalence of PA lesions in root-treated teeth using CBCT images, Nascimento et al. [20] concluded that the prevalence of PA lesions was higher in maxillary molars and anterior teeth [20]. In the current research, we assessed the topography of PA lesions associated with maxillary and mandibular first molars using three orthogonal planes of CBCT slices. Our findings indicated that, overall, the highest mean extension of lesions in this dental group was observed in the vertical dimension, followed by the buccolingual and, lastly, mesiodistal aspects. Other studies have yielded similar findings regarding the inflammatory PA extension patterns in anterior and premolar teeth [21, 22]. These results suggest that, in various regions of the maxillary and mandibular arch, the greatest bone loss primarily occurs in the vertical dimension, followed by the buccolingual and mesiodistal dimensions.

When evaluating the impact of gender on the pattern and amount of lesion extension in the molar region, no statistically significant differences were found between males and females. However, in the anterior region of the jaws, unlike the premolar and molar regions, a significant disparity between genders was observed [22]. Men exhibited the most substantial bone loss in the vertical dimension, while women primarily experienced it in the buccolingual dimension.

Our findings also indicate that men have greater overall bone loss compared to women across all three dental regions [21, 22]. Additionally, alveolar cortical bone thickness and density are consistently higher in males than females, with these differences being more pronounced in the mandible compared to the maxilla, the posterior region as opposed to the anterior, and on the oral side relative to the buccal side [23]. Various factors in combination may contribute to the divergence in bone destruction patterns between males and females in the anterior region.

Bone density decreases with age, with a more pronounced decrease in women due to factors such as female hormones, low bone mass, and longer mean age compared to men [24, 25].

Florenzano et al. [26] noted that the amount of bone regeneration is related to age, with its quantity and quality changing over time. In terms of patient age, the pattern of bone destruction in the anterior, premolar, and molar regions appears consistently across various age groups. Concerning patient age, the pattern of bone destruction in the three tooth groups (anterior, premolar, and molar regions) appears to be consistent across various age groups [21, 22]. However, PA lesions tend to be more extensive in younger patients compared to older ones. Younger patients with inflammatory PA lesions often experience more significant bone destruction. The present study found a statistically significant difference in lesion extension between the 14−44-year-old group and the 55−77-year-old group in the vertical and mesiodistal aspects. The pattern of bone destruction was consistent in both the maxilla and mandible, aligning with previous research on the anterior and premolar regions [21, 22]. However, earlier studies reported greater bone loss in the maxilla for the anterior region and in the mandible for the premolar region [21, 22]. Our study revealed a higher level of bone destruction associated with molar teeth in the mandible compared to the maxilla. The thickness of spongy bone, which differs between the maxilla and mandible, significantly impacts lesion extension in these dental arches [23].

Additionally, the mesiobuccal root of the first molar exhibited the highest mean lesion extension in the vertical aspect.

## 5. Conclusion

Considering the constraints of the current study, the PA lesion extension within the bony structure of the maxilla and mandible appears to adhere to a specific pattern. Considering this, future research endeavors should encompass diverse communities and ethnicities, as well as explore variations in lesion volume. Furthermore, it is worth emphasizing that the CBCT method stands out as a dependable imaging approach for assessing the morphology and morphometric aspects of PA lesion extension and topography.

## Figures and Tables

**Table 1:** Mean (SD) lesion extension in three aspects of the samples under study in terms of age.

Lesion extension aspect	14–44-year-old group		45–54-year-old group		55–77-year-old group		Total		*p*-Value
	Frequency	Mean (SD) (mm)	Frequency	Mean (SD) (mm)	Frequency	Mean (SD) (mm)	Frequency	Mean (SD) (mm)	
Occlusoapical aspect	97	2.25 (1.79)	51	2.2 (1.53)	49	1.46 (1.15)	197	2.04 (1.61)	0.015
Mesiodistal aspect	97	1.91 (1.32)	51	1.77 (1.07)	49	1.38 (0.97)	197	1.74 (1.19)	0.044
Buccolingual aspect	97	2.00 (1.43)	51	1.87 (1.12)	49	1.50 (1.04)	197	1.84 (1.28)	0.082

**Table 2:** Mean (SD) lesion extension in three aspects of the samples under study in terms of gender.

Lesion extension aspect	Males		Females		Total		*p*-Value
	Frequency	Mean (SD) (mm)	Frequency	Mean (SD) (mm)	Frequency	Mean (SD) (mm)	
Occlusoapical aspect	84	2.14 (1.68)	113	1.96 (1.57)	197	2.04 (1.61)	0.439
Mesiodistal aspect	84	1.94 (1.31)	113	1.77 (1.25)	197	1.84 (1.28)	0.721
Buccolingual aspect	84	1.77 (1.16)	113	1.71 (1.22)	197	1.74 (1.19)	0.353

**Table 3:** Mean (SD) lesion extension in three aspects of the samples under study in terms of the dental arch.

Lesion extension aspect	Maxilla		Mandible		Total		*p*-Value
	Frequency	Mean (SD) (mm)	Frequency	Mean (SD) (mm)	Frequency	Mean (SD) (mm)	
Occlusoapical aspect	121	1.91 (1.53)	76	2.25 (1.73)	197	2.04 (1.61)	0.166
Mesiodistal aspect	121	1.67 (1.20)	76	1.85 (1.18)	197	1.74 (1.19)	0.284
Buccolingual aspect	121	1.72 (1.22)	76	2.03 (1.35)	197	1.84 (1.28)	0.110

**Table 4:** Mean lesion extension in three aspects in the samples under study in terms of tooth root type.

Lesion extension aspect	Distal root		Mesial root		Distobuccal root		Mesiobuccal root		Palatal root		Total		*p*-Value
	Frequency	Mean (SD) (mm)	Frequency	Mean (SD) (mm)	Frequency	Mean (SD) (mm)	Frequency	Mean (SD) (mm)	Frequency	Mean (SD) (mm)	Frequency	Mean (SD) (mm)	
Occlusoapical aspect	40	3.16 (2.36)	64	3.63 (2.27)	40	2.99 (1.76)	95	3.85 (2.16)	50	3.11 (2.19)	289	3.52 (2.18)	0.356
Mesiodistal aspect	40	3.17 (1.74)	64	3.04 (1.72)	40	2.48 (1.58)	95	3.17 (1.72)	50	2.93 (1.70)	289	3.01 (1.70)	0.645
Buccolingual aspect	40	3.22 (1.52)	64	3.50 (1.80)	40	2.99 (1.76)	95	3.40 (1.80)	50	2.85 (1.90)	289	3.16 (1.75)	0.024

## Data Availability

All data analyzed during this study are included in this published article. If any, additional data/files may be obtained from the corresponding author upon reasonable request.
